# Case Report: High-dose immunoglobulins prior to plasma exchange in severe pulmonary renal syndrome

**DOI:** 10.3389/fimmu.2023.1210321

**Published:** 2023-06-09

**Authors:** Ann-Kathrin Schäfer, Sascha Dierks, Moritz Schnelle, Peter Korsten, Samy Hakroush, Björn Tampe

**Affiliations:** ^1^ Department of Nephrology and Rheumatology, University Medical Center Göttingen, Göttingen, Germany; ^2^ Institute of Clinical Chemistry, University Medical Center Göttingen, Göttingen, Germany; ^3^ Institute of Pathology, University Medical Center Göttingen, Göttingen, Germany

**Keywords:** ANCA-associated vasculitis, pulmonary renal syndrome, IVIGs, plasma exchange, ANCA clearance

## Abstract

Plasma exchange rapidly depletes pathogenic anti-neutrophil cytoplasmic autoantibodies (ANCAs) and is considered for induction therapy in severe ANCA-associated vasculitis. The aim of plasma exchange is to remove putative disease mediators from the circulation, such as toxic macromolecules and pathogenic ANCAs. To our knowledge, we here provide the first report of applying high-dose IVIGs prior to plasma exchange and assessment of ANCA autoantibody elimination in a patient with severe pulmonary renal syndrome due to ANCA-associated vasculitis. After high-dose application of intravenous immunoglobulins (IVIGs) prior to plasma exchange treatment, efficacy of myeloperoxidase (MPO)-ANCA autoantibody elimination was substantially increased, associated with rapid clearance of MPO-ANCA autoantibodies. High-dose IVIGs resulted in marked reduction of MPO-ANCA autoantibody levels and did not directly affect autoantibody clearance by plasma exchange itself, as also confirmed by comparable MPO-ANCAs in the exchange fluid relative to serum levels. Moreover, measurements of serum creatinine and albuminuria confirmed that high-dose IVIGs were well tolerated and did not exacerbate kidney injury.

## Introduction

The field of anti-neutrophil cytoplasmic antibody (ANCA)-associated vasculitis (AAV) has seen considerable progress in understanding of the complexity of pathophysiology, the course of the diseases and importantly therapeutic options, that have been translated into an improved patient survival in the most contemporary cohorts. Plasma exchange (PEX) rapidly depletes pathogenic ANCA autoantibodies and is considered for induction therapy in severe AAV (1). This is particularly important in patients presenting with severe diffuse pulmonary hemorrhage (DAH) that is immediately life threatening, where rapid ANCA clearance might make a “life or death” difference. The PEXIVAS trial did not find significant survival benefits for PEX in AAV, there is data supporting PEX may reduce the acute mortality of patients presenting with severe DAH, especially in those with MPO-ANCA positivity (2–4). Therefore, PEX is often recommended as an adjunctive therapy in severe cases of AAV presenting with rapidly progressive glomerulonephritis (RPGN) and/or DAH (5). In this context, it is attractive to speculate that strategies to enhance clearance of pathogenic ANCA autoantibodies might be beneficial especially in severe cases of AAV. Among them, efficacy of high-dose intravenous immunoglobulins (IVIGs) to decrease circulating ANCA autoantibody levels in AAV patients has already been shown (6, 7). However, direct assessment of efficacy to eliminate ANCA autoantibodies by PEX combined with IVIGs pretreatment in AAV has not been assessed yet. We here pursued an ANCA level-driven approach to determine efficacy of ANCA autoantibody clearance by PEX and additional high-dose IVIGs in severe pulmonary renal syndrome due to AAV.

## Treatment protocols

The patient received a therapy regime consisting of an immunosuppressive therapy with prednisolone and intravenous cyclophosphamide (CYC, 10 mg/kg body weight) according to the CYCLOPS protocol (8). Additional PEX was performed on alternate days with a total plasma volume of 3000 mL using the Plasauto ∑ (Asahi Kasei Medical Co., Ltd., Tokyo, Japan) and Plasmaflo OP-08W(L) filter (Asahi Kasei Medical Co., Ltd., Tokyo, Japan) according to the manufacturer’s protocol, low-dose unfractionated heparin was used only during PEX treatment. Since the patient suffered from pulmonary hemorrhage, separated plasma was substituted by fresh frozen plasma (FFP) during all PEX treatments. After premedication with 2 mg clemastine, IVIGs at a dosage of 1 g/kg body weight (70 g in total, Kiovig 10g/100mL, Takeda Manufacturing Austria AG, Vienna, Austria) were infused two hours prior to PEX treatments.

## Measurements of ANCA autoantibodies

Assessment of serum levels for myeloperoxidase (MPO)- and proteinase 3 (PR3)-ANCAs was performed using immunoassays (ImmunoCAP 250, Thermo Fisher Scientific, Waltham, USA). ANCA immunofluorescence was conducted according to the manufacturer’s protocol (EUROIMMUN AG, Lübeck, Germany).

## Data analyses

Data analyses were performed with GraphPad Prism (version 9.3.1 for macOS, GraphPad Software, San Diego, California, USA).

## Case report

**Figure 1:**
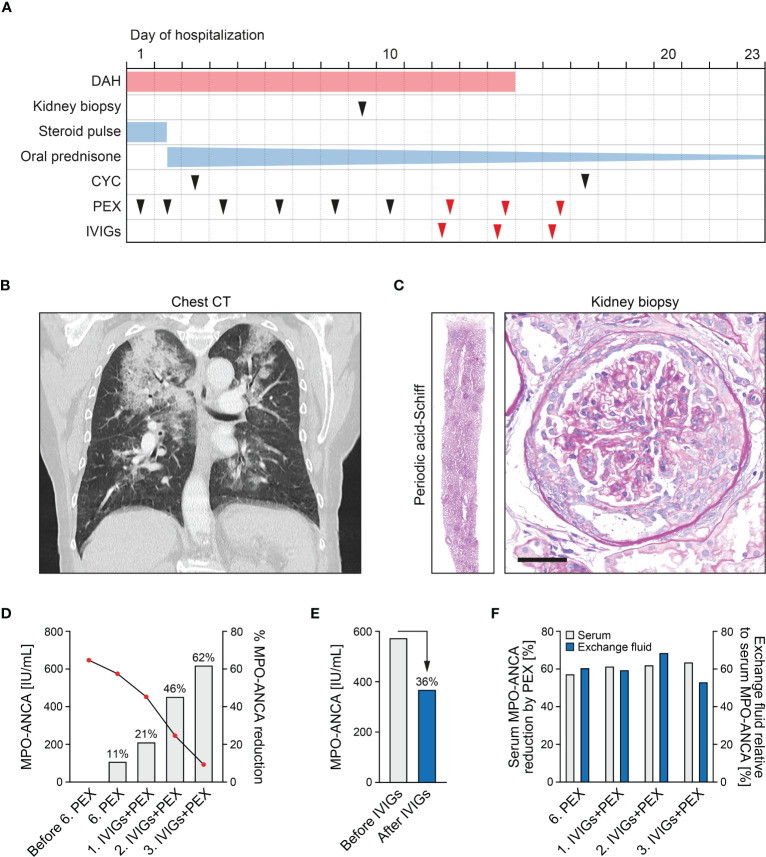
Efficacy of ANCA autoantibody clearance by high-dose IVIGs prior to PEX treatment. **(A)** Timeline of the reported case with clinical symptoms, diagnostic workup and treatment regimens. **(B)** Chest CT scans of the lungs showed bilateral ground-glass opacities and severe DAH compatible with pulmonary vasculitis. **(C)** A representative kidney section stained with periodic acid-Schiff confirmed mixed class pauci-immune, necrotizing, and crescentic ANCA-associated glomerulonephritis (scale bar: 50 μm). **(D)** Measurements of MPO-ANCAs are aligned to the left axis, bar graphs of percentual MPO-ANCA reduction without and with high-dose application of IVIGs (1 g/kg body weight) two hours prior to PEX treatment to the right axis. **(E)** Measurements of serum MPO-ANCA autoantibody levels before/after the first application of high-dose IVIGs. **(F)** Measurements of percentual serum MPO-ANCA reduction directly assessed before/after PEX treatment are aligned to the left axis, percentual MPO-ANCAs measured in the exchange fluid relative to serum levels to the right axis.

**Figure 2:**
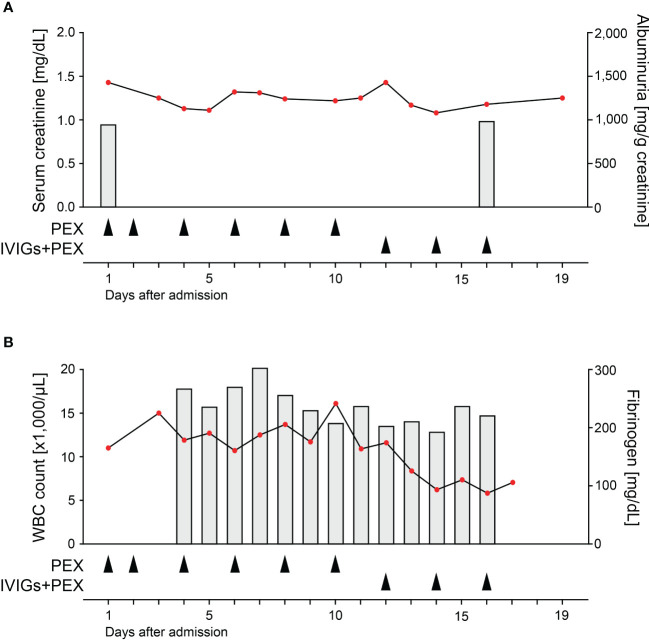
High-dose IVIGs did not exacerbate kidney injury. **(A)** Measurements of serum creatinine are aligned to the left axis, bar graphs of albuminuria to the right axis. Respective arrowheads indicate PEX without and with high-dose application of IVIGs (1 g/kg body weight) two hours prior to treatment. **(B)** Measurements of WBC counts are aligned to the left axis, bar graphs of fibrinogen levels to the right axis. Respective arrowheads indicate PEX without and with high-dose application of IVIGs (1 g/kg body weight) two hours prior to treatment.

A 75-year-old woman without pre-existing illnesses was referred to our department. Four days prior, she presented to an external hospital with arthralgia and developed acute haemoptysis, a bronchoscopy confirmed severe DAH in both upper lobes. At admission, she presented with lung failure requiring non-invasive mechanical ventilation (PaO_2_/FiO_2_ ratio 153 mmHg), acute kidney injury (serum creatinine of 1.43 mg/dL, reference range: 0.5-1 mg/dL; eGFR 36 mL/min/1.73 m^2^, reference range: >60 mL/min/1.73 m^2^), and albuminuria (953 mg/g creatinine, reference range: <30 mg/g), and hematuria (**Figure 1A** and **Table 1**). Computed tomography (CT) scans showed bilateral ground-glass opacities and severe DAH compatible with pulmonary vasculitis (**Figure 1B**), a kidney biopsy showed pauci-immune crescentic ANCA-associated glomerulonephritis (**Figure 1C**), and laboratory testing confirmed presence of myeloperoxidase (MPO)-ANCA autoantibodies (>134 IU/mL, reference range: <3.5 IU/mL, **Table 1**). Based on the diagnosis of life threatening pulmonary renal syndrome due to AAV, steroid pulse, intravenous cyclophosphamide (CYC, 10 mg/kg body weight) according to the CYCLOPS protocol, and additional PEX treatment (total plasma volume of 3000 mL, Plasauto ∑ and Plasmaflo OP-08W(L) filter, Asahi Kasei Medical Co., Ltd., Tokyo, Japan) were initiated (8). After a total number of six PEX treatments, pulmonary hemorrhage was still present requiring non-invasive mechanical ventilation (PaO_2_/FiO_2_ ratio 155 mmHg), and a repeated testing for MPO-ANCAs still confirmed high levels outside of the upper range (>134 IU/mL). Direct assessment of MPO-ANCAs before the sixth PEX treatment and two days thereafter showed limited efficacy of circulating MPO-ANCA autoantibody elimination (647 IU/mL to 575 IU/mL, 11% reduction, **Figure 1D**). After high-dose application of IVIGs (1 g/kg body weight, 70 g in total, Kiovig 10g/100mL, Takeda Manufacturing Austria AG, Vienna, Austria) two hours prior to the next PEX treatment, efficacy of MPO-ANCA autoantibody elimination was almost doubled (575 IU/mL to 452 IU/mL, 21% reduction, **Figure 1D**). Based on these observations, IVIGs were administered prior to additional two PEX treatments and resulted in rapid clearance of MPO-ANCA autoantibodies (**Figure 1D**). High-dose IVIGs resulted in marked reduction of MPO-ANCA autoantibody levels (575 IU/mL to 370 IU/mL, 36% reduction, **Figure 1E**) and did not directly affect autoantibody clearance by PEX itself, as also confirmed by comparable MPO-ANCAs in the exchange fluid relative to serum levels (**Figure 1F**). Pulmonary hemorrhage rapidly ceased and mechanical ventilation was no longer required, while high-dose IVIGs were well tolerated not exacerbating kidney injury (**Figure 2A**). Furthermore, white blood cell (WBC) counts (reference range: 4,000-11,000/µL), and fibrinogen levels (reference range: 200-393 mg/dL) were stable by using FFP substitution (**Figure 2B**). During the half year follow-up, the patient finished remission induction therapy with CYC and is currently receiving maintenance therapy with rituximab (RTX). Kidney function recovered (serum creatinine of 0.92 mg/dL, reference range: 0.5-1 mg/dL; eGFR 61 mL/min/1.73 m^2^, reference range: >60 mL/min/1.73 m^2^, albuminuria 273 mg/g creatinine, reference range: <30 mg/g). In summary, we here report an enhanced ANCA autoantibody clearance by high-dose IVIGs prior to plasma exchange in a case of severe pulmonary renal syndrome.

**Table 1:** Laboratory parameters at admission.

Parameter	Value	Normal range
Hemoglobin – g/dL	8.8	13.5-17.5
WBC count – 1,000/µL	11	04-Nov
INR – ratio	1.2	0.8-1.2
aPTT – seconds	26	25-37
AST – U/L	17	≤35
ALT – U/L	9	≤45
AP – U/L	57	40-150
GGT – U/L	19	Dec-64
Haptoglobin – g/L	3.21	0.14-2.58
LDH – U/L	232	125-250
Total bilirubin – mg/dL	<0.3	0.3-1.2
Creatinine – mg/dL	1.31	0.7-1.2
eGFR – mL/min/1.73 m^2^	40	>60
BUN – mg/dL	38	Oct-20
uACR – mg/g creatinine	953	<30
Rheumatoid factor – IU/mL	<10	<15.9
Complement C3c – g/L	1.07	1.07
Complement C4 – g/L	0.19	0.19
Anti-GBM – U/mL	<0.8	<7
ANCA-IF – titer	0.736111111	<1:10
PR3-ANCA – IU/mL	0.4	<2
MPO-ANCA – IU/mL	>134	<3.5
ENA screen – IU/mL	0.1	<0.7
ANA-IF – titer	Pos	Neg
LF-lactoferrin – titer	Neg	Neg
Elastase – titer	Neg	Neg
Catepsin – titer	Neg	Neg
BPI – titer	Neg	Neg
Anti-ds-DNA – IU/mL	15	<15
DSF70 – IU/mL	<0.6	<7
IgA – g/L	2.13	0.63-4.84
IgG – g/L	9.5	5.4-18.2
IgM – g/L	0.72	0.22-2.93

ANA, antinuclear antibody; ANCA, antineutrophil cytoplasmic antibody; anti-GBM, anti-glomerular basement membrane; BPI, bactericidal permeability increasing protein; BUN, blood urea nitrogen; ALT, alanine transaminase; AP, alkaline phosphatase; aPTT, activated partial thromboplastin time; AST, aspartate aminotransferase; ds-DNA, double stranded-DNA; DSF70, dense-fine-speckled 70; ENA, extractable nuclear antigen; GGT, gamma-glutamyl transferase; IgA, immunoglobulin A; IF, immunofluorescence; IgG, immunoglobulin G; IgM, immunoglobulin M; INR, international normalized ratio; LDH, lactate dehydrogenase; MPO-ANCA, myeloperoxidase-ANCA; Neg, negative; Pos, positive; PR3-ANCA, proteinase 3-ANCA, uACR, urinary albumin-to-creatinine ratio; WBC, white blood cell.

## Discussion

On a mechanistic level, ANCA autoantibodies are capable to activate neutrophils and immunization of mice with ANCA autoantibodies purified from AAV patients induce systemic vasculitis (9, 10). These observations support the pathogenic role of ANCA autoantibodies in AAV pathophysiology. Every antibody that is induced and specific for an antigen termed “Ab1” antibody has immunogenic regions, particularly in their unique variable-region antigen-binding domains. High-dose IVIGs a capable to compete with pathogenic IgGs for activating FcγRs, thereby displacing autoantibodies from tissue-bound antigens into the circulation (11). In addition, the immunogenic amino acid sequences called idiotopes of Ab1 antibodies can induce specific antibodies against Ab1 antibodies. The paratopes (or antigen-binding domains) of some of the resulting anti-idiotype “Ab2” antibodies (anti-id) are specific for Ab1 and interact with the variable region of antibodies. Interestingly, it has already been shown that IVIGs contain anti-id capable to neutralize ANCA binding to the respective autoantigen (12). Our observation that IVIGs rapidly decreased ANCA autoantibody levels and did not directly enhance efficacy of PEX itself could implicate formation of ANCA/anti-id complexes and subsequent endogenous clearance. In addition, these antibody complexes might not be accessible for immunoassay measurements. We are aware that our observations require validation in larger study populations, that kinetics may have also been affected by previous induction therapy, pretreatment time of IVIGs or ANCA autoantibody subtypes, and that the exact mechanisms contributing to our observations require further investigation. Furthermore, the immunoassay results were not reliable as values were outside of the manufacturer’s reference range. Nevertheless, this ANCA level-driven approach might contribute to novel therapeutical strategies to increase efficacy of pathogenic ANCA autoantibody clearance in severe AAV.

## Data availability statement

The original contributions presented in the study are included in the article/supplementary material. Further inquiries can be directed to the corresponding author.

## Ethics statement

Ethical review and approval was not required for the study on human participants in accordance with the local legislation and institutional requirements. The patients/participants provided their written informed consent to participate in this study. Written informed consent was obtained for the publication of this case report.

## Author contributions

BT conceived the report, analyzed data and wrote the manuscript. A-KS collected data and revised the manuscript. SD and MS performed ANCA autoantibody measurements, PK was directly involved in patient treatment, and SH performed histological analysis. All authors contributed to the article and approved the submitted version.
